# Reliability and validity of the SF-36 Health Survey Questionnaire in patients with brain tumors: a cross-sectional study

**DOI:** 10.1186/s12955-017-0665-1

**Published:** 2017-05-04

**Authors:** Adomas Bunevicius

**Affiliations:** Neuroscience Institute, Lithuanian University of Health Sciences, Eiveniu g. 2, LT-50009 Kaunas, Lithuania

**Keywords:** Brain tumor, Quality of life, Validity, Reliability, Cancer, Oncology

## Abstract

**Background:**

Deterioration of health related quality of life (HRQoL) is common in brain tumor patients. This study evaluated validity and reliability of the Medical Outcomes Study 36-Item Short Form (SF-36) in patients with brain tumors.

**Methods:**

Two hundred and seventy-seven patients admitted for brain tumor surgery were evaluated for HRQoL (SF-36 questionnaire); depressive symptoms (Beck Depression Inventory-II or BDI-II); and functional status (Barthel index or BI). Final histological diagnosis was obtained from pathology reports.

**Results:**

Two-hundred and twenty-seven (completion rate of 82%) patients (69% women; mean age 55.8 ± 14.4 years) completed the SF-36 questionnaire. The most common brain tumor diagnosis was meningioma (40%), followed high-grade glioma (19%). Missing data rates were ≤4%. Internal consistency was adequate for all (Cronbach α ≥ .728) but Social Functioning (Cronbach α = .527) and General Health (Cronbach α = .693) subscales. Ceiling (≥36%) and floor (≥22%) effect rates were the greatest for the Role Limitations subscales. The SF-36 subscales pertaining physical health correlated the strongest with the BI score, while the SF-36 subscales pertaining emotional health correlated the strongest with the BDI-II score. Patients with mild-moderate depressive symptoms (BDI-II score ≥20) scored lower across all SF-36 subscales, and handicap patients (BI score <90) scored the lower across all, but Mental Health, subscales.

**Conclusions:**

The SF-36 is a valid and reliable instrument in brain tumor patients and therefore can be reliably applied for evaluation of HRQoL in neuro-oncology setting. Further studies exploring other psychometric properties of the SF-36 in brain tumor patients across disease progression stages are warranted.

## Background

Health-related quality of life (HRQoL) is commonly used as an outcome measure [1–3] and has prognostic significance in brain tumor patients. For example, improved HRQoL is associated with longer survival of high-grade glioma patients [4] while poor HRQoL predicts shorter survival of low-grade glioma patients [5].

Numerous multi-item questionnaires are used for evaluation of HRQoL in patients with brain tumors [6]. The Medical Outcomes Study 36-Item Short Form (SF-36) health survey comprehensively evaluates patient perceived health status across broad physical and emotional health domains [7]. It is the most commonly used generic HRQoL assessment tool worldwide, including patients with cancer [7, 8]. Due to extensive use and experience across patients and general population, the generic SF-36 questionnaire also allows to compare patient perceived health status across a variety of disorders and with the general population. Adequate psychometric properties of the SF-36 were documented in cancer patients [9] and in cancer survivors [10]. However, there are no studies specifically evaluating reliability and validity of the SF-36 questionnaire in patients with established brain tumor diagnosis.

The goal of the study was to evaluate reliability and validity of the SF-36 questionnaire in patients diagnosed with brain tumors.

## Methods

### Patients

Consecutive adult patients admitted for surgery of primary and metastatic brain tumors at the Department of Neurosurgery of Hospital of Lithuanian University of Health Sciences, Kaunas, Lithuania, in a period from March 2010 until June 2011 were invited in the study. Patients who were unable to speak Lithuanian, or with severe cognitive or neurologic deficits were excluded.

### Data collection

The study was approved by the Ethics Committee for Biomedical Research at the Lithuanian University of Health Sciences, Kaunas, Lithuania. All patients gave signed informed consent.

All study patients were approached within 3 days before elective brain tumor surgery and were evaluated for sociodemographic characteristics (age, gender, highest educational degree and marital status) and previous brain tumor treatments. During the same visit patients were evaluated for HRQoL (SF-36 questionnaire [7]) and depressive symptom severity (Beck Depression Inventory-II (BDI-II) [11]) Patients were asked to complete the SF-36 and BDI-II questionnaires themselves and were given an opportunity to ask questions if any. Functional status was evaluated by the study investigator during the same visit using the Barthel index (BI) [12]. The BDI-II and BI were selected to determine construct validity of the SF-36.

Brain tumor diagnoses were obtained from the final pathology reports. Patients diagnosed with high-grade glioma and metastatic tumor were categorized as having malignant brain tumor.

### Questionnaires

The SF-36 questionnaire includes eight multiple-item subscales that evaluate physical function, social functioning, role limitations due to physical problems, role limitations due to emotional problems, mental health, vitality, pain, and general health perception [7]. Total score on each SF-36 subscale ranges between 0 and 100. Greater score indicates better HRQoL.

BDI-II has 21 items designed to evaluate depressive symptom severity during the previous 2 weeks [11]. Total BDI-II score ranges between 0 and 63 with greater score indicating more severe depressive symptoms. Patients were dichotomized as having minimal (BDI-II score <20) or moderate to severe (BDI-II score ≥20) depressive symptoms. Scores on the BDI-II items 12 (interest in other people) and 17 (fatigue) were selected to evaluate convergent validity of the SF-36 subscales of Social Functioning and Vitality, respectively. Examination of individual BDI-II items is recommended for more detailed assessment of patients’ clinical presentation [13]. Adequate construct validity and psychometric properties of the Lithuanian translation of the BDI-II were previously documented in patients with brain tumors [14].

Functional status was measured with the BI [12]. Total BI score ranges from 0 to 100, with lower score indicating greater functional impairment. Patients were dichotomized as functionally dependent (BI score <90) or as functionally independent (BI score ≥90). The BI is a commonly used instrument for evaluation of functional status in hospital setting in Lithuania [3].

### Statistical analyses

Data were analyzed using the PASW for Windows (IBM Corporation, Chicago, Illinois). Two-tailed probability values of <0.05 were considered as statistically significant.

Age, gender, brain tumor grade, and proportion of functionally dependent and moderately-to-severely depressed patients were compared in responders vs. non-responders using Pearson *X* ^*2*^ test and Mann-Whitney test. Internal consistency of the SF-36 subscales was evaluated using the Cronbach’s coefficient α. Internal consistency was considered adequate if Cronbach’s coefficient α values were >0.70 [15, 16]. Floor and ceiling effects for each SF-36 subscale were defined as the proportion of patients who achieved the lowest or the highest possible score, respectively, on each subscale. Floor and ceiling effect was considered present if at least 15% of respondents reached the lowest or the highest possible score, respectively [16].

Convergent validity was measured by calculating the Spearman correlation coefficient of all SF-36 subscale scores with scores on the BI, SF-36 Physical and Mental component summary, BDI-II total, and BDI-II items 12 (interest in other people) and 17 (fatigue). It was expected that scores on the SF-36 subscales pertaining to mental health would correlate the strongest with the SF-36 Mental component summary score and BDI-II score, while sores on the SF-36 subscales pertaining to physical health would correlate the strongest with the SF-36 Physical component summary score and BI score. Furthermore, strong correlation was expected between the SF-36 Social Functioning and Vitality scores with scores on the BDI-II items 12 (interest in other people) and 17 (fatigue), respectively. Construct validity of the SF-36 was evaluated by comparing SF-36 scores as a function of tumor grade (malignant brain tumor vs. not-malignant tumor), patient age (≥50 years vs. <50 years), depressive symptom severity (moderate-severe vs. minimal-mild depressive symptoms) and functional status (functionally dependent vs. independent) using Mann-Whitney test. It was hypothesized that greater age, diagnosis of malignant brain tumor, moderate to severe depressive symptom severity and functional dependence would be associated with lower physical and mental component scores of the SF-36.

## Results

Two hundred and seventy-seven patients were administered the battery of questionnaires and 227 (response rate of 82%) patients completed the SF-36 questionnaire (Table 1). The proportion of patients with moderate-severe depressive symptoms was lower in responders vs. non-responders (0% vs. 19%, *p* = 0.01). There were no statically significant differences between responders and non-responders in age (*p* = 0.334), gender (*p* = 0.243) brain tumor grade (*p* = 0.928) and BI score (*p* = 0.271). The majority of the study patients were women (69%). Mean age of patients was 55.8 ± 14.4 years. The BDI-II was completed by 203 (89%) patients and BI was administered to 213 (94%) patients. The most common brain tumor diagnosis was meningioma (40%), followed high-grade glioma (19%).

**Table 1:** Demographic and clinical characteristic of the study patients

Characteristic	Mean ± SD, number (%)
Age (years)	55.8 ± 14.4
Gender	
Men	70 (31%)
Women	157 (69%)
Highest education degree	
Some high-school	27 (12%)
High-school graduate	96 (42%)
Some university education	57 (25%)
University degree	47 (21%)
Marital status	
Single	34 (15%)
Married	151 (67%)
Divorced	18 (8%)
Widower	22 (10%)
Brain tumor diagnosis	
High-grade glioma	44 (19%)
Low-grade glioma	19 (8%)
Meningioma	91 (40%)
Pituitary adenoma	27 (12%)
Vestibular schwanoma	20 (9%)
Metastatic tumor	2 (1%)
Other	24 (10%)
Recurrent disease	37 (16%)
Beck Depression Inventory – II	
Score	11.38 ± 9.80
Minimal to mild depressive symptoms	190 (84%)
Moderate to severe depressive symptoms	37 (16%)
Barthel Index	
Score	96.22 ± 10.43
Functionally dependent	15 (7%)
Functionally independent	212 (93%)

**Fig. 1:**
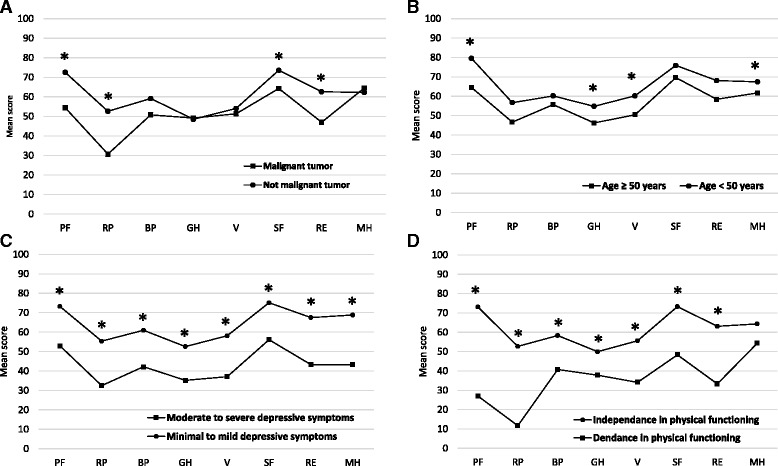
Mean scores on the SF-36 subscales as a function of tumor grade (**a**), age (**b**), depressive symptom severity (**c**) and functional dependence (**d**). PF, Physical Function; RP, Role Limitations Due to Physical Problems; BP, bodily pain; GH, General Health; V, Vitality; SF, Social Functioning; RE, Role Limitations Due to Emotional Problems; MH, mental Health

Mean scores on the SF-36 subscales are presented in Table 2. Missing data rates were low (≤4%) for all subscales. The lowest SF-36 scores were for the General Health and Role Limitations Due To Physical Problems subscales while the highest scores were for the Social Functioning and Physical Functioning subscales. Cronbach’s coefficients α were greater than .70 for all but General Health (.693) and Social Functioning (.527), subscales. Ceiling effect was present for the SF-36 subscales of Role Limitations Due To Emotional Problems (51%), Role Limitations Due To Physical Problems (36%), Social Functioning (27%) and Pain (23%) subscales. Floor effect was present for the SF-36 subscales of Role Limitations Due To Physical Problems (26%) and Role Limitations Due To Emotional Problems (22%). As expected, BI and SF-36 Physical component summary scores correlated stronger with the SF-36 subscales pertaining to physical health (Spearman rho values range from .269 to .487 and from .542 to .829, respectively) relative to the SF-36 subscales pertaining to emotional health (Spearman rho values range from .235 to .311 and from .539 to .643, respectively) (Table 3). Alternatively, the SF-36 Mental component summary score correlated stronger with the SF-36 subscales pertaining to emotional health (Spearman rho values range from .746 to .820) than with the SF-36 subscales pertaining to physical health (Spearman rho values range from .486 to .682). The BDI-II total scores correlated the strongest with the SF-36 subscales of Vitality (rho = −.602) and Mental Health (rho = −.601). BDI-II items evaluating social interactions and fatigue correlated the strongest with the SF-36 Mental Health subscale followed by the SF-36 Social Functioning (rho = −.463) and Vitality (−.440) subscales, respectively. Known-group comparison analyses demonstrated the expected findings, i.e., more advanced age, diagnosis of malignant brain tumor, moderate to severe depressive symptom severity and functional dependence were associated with lower SF-36 scores, indicating adequate discriminative abilities of the SF-36 (Fig. 1). Malignant brain tumor patients scored significantly lower on the SF-36 Physical Functioning (*p* = 0.001), Social Functioning (*p* = 0.042) and Role Limitations Due To Physical Functioning (*p* = 0.002) and Emotional Functioning (*p* = 0.034) subscales relative to patients with not malignant brain tumors. Patients aged ≥50 years scored significantly lower on the SF-36 Physical Functioning (*p* < 0.001), General Health (*p* = 0.001), Vitality (*p* = 0.002) and Mental Health (*p* = 0.04) subscales relative to patients < 50 years old. Mild to moderate depressive symptoms were associated with lower scores on all SF-36 subscales (all *p*-values ≤0.006) and functional dependence was associated with lower scores on all (all *p*-values <0.004) but Mental Health subscale of the SF-36.

**Table 2:** SF-36 scores and internal consistency

SF-36 subscales	No of items	Mean ± SD	Median [IQR]	Ceiling %	Floor %	Missing %	Cronbach α
Physical Functioning	10	69.28 ± 27.28	80 [38]	6	3	1	.924
Role Limitations Due To Physical Problems	4	49.89 ± 42.95	50 [100]	36	26	1	.873
Role Limitations Due To Emotional Problems	3	61.48 ± 43.50	100 [100]	51	22	1	.862
Vitality	4	53.47 ± 21.37	55 [30]	1	0	4	.728
Emotional Well-Being	5	63.49 ± 19.57	64 [28]	0	0	4	.766
Social Functioning	2	71.61 ± 25.69	75 [50]	27	0	3	.527
Pain	2	57.09 ± 31.29	52 [53]	23	2	3	.836
General Health	5	48.94 ± 20.06	50 [27]	0	1	4	.693

*IQR* interquartile range

**Table 3:** Convergent validity of the SF-36 questionnaire^a^

SF-36 subscales	Barthel index	SF-36 physical component summary	SF-36 mental component summary	Beck depression Inventory-II		
				Total score	Item 12: interest in other people	Item 17: fatigue
Physical Functioning	.487	.755	.541	-.519	-.298	-.206
Role Limitations Due To Physical Problems	.246	.829	.682	-.376	-.212	-.162
Pain	.296	.718	.486	-.342	-.250	-.281
General Health	.269	.542	.537	-.519	-.415	-.342
Vitality	.311	.643	.746	-.602	-.413	-.440
Social Functioning	.277	.643	.749	-.476	-.463	-.306
Role Limitations Due To Emotional Problems	.235	.607	.820	-.355	-.258	-.143
Mental Health	.239	.539	.752	-.601	-.488	-.466
Mental component summary	.329	.769	-	-.606	-.455	-.354
Physical component summary	.429	-	.769	-.564	-.356	-.317

^a^Spearman rank-order correlation

All *p*-values <0.05

## Discussion

The study findings indicate that the SF-36 is a reliable and valid instrument for assessment of HRQoL in patients with brain tumors.

The proportion of missing data was low (≤4%) for all SF-36 subscales corresponding to previous studies in healthy participants and in patients with somatic disorders. For example, missing data rates were comparable in the original Medical Outcomes Study from the United States (range from 1.1 to 5.9%) [17] and in a large sample (*n* = 6822) of Dutch healthy participants and patients with migraine and various cancers (range from 1 to 6.6%) [18]. Ceiling and floor effects were present for the Role Limitations and Emotional Functioning subscales indicating low sensitivity of the SF-36 to monitor subtle variations of functional status and emotional functioning in brain tumor patients. High ceiling effect rate can be explained by dichotomous answers to the SF-36 questionnaire items on the respective subscales. Higher ceiling effect (76%) of the SF-36 Physical and Emotional Role Limitations subscales were previously reported in childhood cancer survivors [10]. Ceiling effect observed for the SF-36 Social Functioning and Bodily Pain subscales indicates that social functioning impairment and pain are not severe symptoms in brain tumor patients. Required threshold rate for floor and ceiling effect was not reached for other SF-36 subscales [16] suggesting adequate sensitivity of the SF-36 for evaluating perceived health status of brain tumor patients.

The SF-36 subscales had adequate internal consistency satisfying the recommended threshold value of .70 [15], with the exception of the General Health and Social Functioning subscale. Low Cronbach’s α values of the SF-36 Social Functioning subscale was previously reported in Spanish patients with coronary artery disease (Cronbach’s α = .45) [19] and in Dutch cancer patients (Cronbach’s α = .50) [18]. Therefore, other instruments can be considered for assessment of social functioning in hospitalized brain tumor patients.

As expected, scores on the SF-36 subscales pertaining to perceived physical and emotional health status demonstrated good agreement with other measures of physical and mental health status, respectively, indicating adequate criterion validity of the SF-36 questionnaire. These findings correspond to previous studies in patients with neurological disorders [20, 21] and non-CNS cancers [9, 10]. Our findings suggest that other instruments assessing general health status and social functioning in in-hospital population of cancer patients can be considered.

The SF-36 demonstrated good discriminate abilities. Patients with malignant brain tumors had worse physical and social functioning and greater role limitations relative to patients with not malignant tumors. Due to progressive and incurable nature, malignant brain tumors are associated with progressive disability [22] and worsening of mental health status [23]. Age of 50 years or greater was associated with worse physical functioning, general health and mental health and with greater fatigue symptom severity, echoing with previous studies demonstrating detrimental effect of age on HRQoL in patients with brain tumors [23, 24]. Moderate-severe depressive symptoms were associated with worse HRQoL across all SF-36 dimensions. Mood symptoms and disorders are common [25–27] and were document as independent predictors of worse HRQoL of brain tumor patients [3, 26]. Functional dependence was associated with worse HRQoL in all but mental health domains suggesting that functional status should be considered important for HRQoL of brain tumor patients. The observed statically significant differences of the SF-36 subscale scores between patient subgroups were also clinically relevant because they exceed a 5 point threshold that was suggested by Ware [28] and is commonly used across patient populations [29]. However, others reported that minimal clinically important thresholds are different across diseases and SF-36 subscales [30]. For example, a study in vestibular schwannoma patients found that the minimal clinically important difference of the SF-36 was 7 points for the Mental Health Component Summary score and 8 points for the Physical Health Component Summary score [31]. Further studies should clarify clinically important thresholds of the SF-36 subscales across brain tumor patients.

Numerous generic and brain tumor specific questionnaires are used for evaluation of HRQoL in patients with brain tumors [6]. Generic HRQoL instruments can allow to evaluate broader domains of impairment imposed by a brain tumor and allow to compare HRQoL across disorders and with the general population. For example, scores on all SF-36 subscales in our cohort of brain tumor patients was lower when comparing to previously published SF-36 scores in the general population [32], indicating that brain tumor is associated with significant impairment of perceived physical and mental health status. On the other hand, generic questionnaires do not collect information on all the areas of well-being and functioning that may be important to patients with brain neoplasms, meaning they may not be sensitive enough to assess changes. The Brain cancer-specific Quality of Life Questionnaire (QLQ-BN20) of the European Organization for Research and Treatment of Cancer (EORTC) was specifically developed to evaluate HRQoL in patients with brain neoplasms [33] and demonstrated adequate cross-cultural psychometric properties. Brevity and disease specificity are the major advantages of the EORTC-BN20 scale. The decision to choose HRQoL instrument should be selected based on clinical and research needs.

Heterogeneous sample in terms of brain tumor histological diagnosis prevented from evaluating psychometric properties of the SF-36 questionnaire in more homogenous group of patients. Results should be generalized with caution to patients at different disease progression and to patients receiving adjuvant therapies (i.e., radiotherapy or chemotherapy) because only patients admitted for brain tumor surgery and not receiving adjuvant therapies were studied. In addition, this setting of recruitment may include a selection bias, because patients hospitalized just before surgery may be stressed and have more complaints, thereby leading to over-representation of severe symptoms. Studies addressing psychometric properties of the SF-36 questionnaire at different stages of disease progress are encouraged.

## Conclusions

This is the first study demonstrating that the SF-36 questionnaire has adequate reliability and validity in patients with brain tumors. The SF-36 questionnaire can be considered for assessment of HRQoL in neuro-oncology setting. Other psychometric properties of the SF-36 questionnaire, including Reliability (test-retest reliability, measurement error), structural validity, responsiveness (smallest detectable change) and interpretability (minimal important change) remain to be addressed in brain tumor patients.

## Abbreviations

BDI-II: Beck Depression Inventory-II
BI: Barthel index
HRQoL: Health related quality of life
SF-36: The medical outcomes study 36-item short form
